# The Effects of a 16-Week Periodized Resistance Training Program on the Strength, Power, and Body Composition of Elite Collegiate Cheerleaders

**DOI:** 10.3390/sports14050177

**Published:** 2026-04-28

**Authors:** Seiichiro Takei, Kei Kato, Mamiko Ichikawa, Kana Iwano

**Affiliations:** Institute of Sports Science & Medicine, Teikyo University, Tokyo 192-0395, Japan; katou.kei.wv@teikyo-u.ac.jp (K.K.); ichikawa.mamiko.mp@teikyo-u.ac.jp (M.I.); iwano.kana.ab@teikyo-u.ac.jp (K.I.)

**Keywords:** cheerleading, strength, power, body composition, periodization, power clean

## Abstract

This study examined the effects of a 16-week periodized resistance training program on the strength, power, and body composition of elite collegiate cheerleaders. Thirteen female athletes from a nationally top-ranked university team completed a structured program comprising hypertrophy and strength/power phases. Performance testing at pre-, mid-, and post-intervention included one-repetition maximum (1RM) assessments for the front squat, power clean, and shoulder press, as well as measurements of body mass, lean body mass, and body fat percentage. All strength measures improved significantly across the intervention (front squat: +14.0%, power clean: +17.7%, and shoulder press: +18.3%). Body fat percentage decreased by 6.7%, and lean body mass increased by 2.6%, while total body mass remained statistically unchanged. These results demonstrate that periodized resistance training can elicit meaningful improvements in performance and body composition without increases in body mass. Moreover, the final post-intervention 1RM values—1.43× body mass for the front squat, 1.11× for the power clean, and 0.73× for the shoulder press—offer preliminary benchmarks for the strength performance of high-level collegiate cheerleaders.

## 1. Introduction

Cheerleading has evolved from its origins in the United States as an organized crowd support activity to a globally recognized competitive sport. Modern cheerleading routines incorporate diverse elements, including arm motions, jumps, tumbling, and partner stunts and pyramids [1]. These stunt movements involve athletes in defined roles: bases who lift and stabilize, tops (also known as flyers) who are elevated, and spotters who assist with safety. In advanced structures, such as three-layer pyramids, a middle layer is added to support the top(s) between the foundational bases.

Athletes in the base, spotter, and middle positions frequently engage in lifting, catching, and stabilizing the top athletes, and as performance demands increase, greater height, control, and stability are warranted. A recent study by Riddell et al. [2] demonstrated that competitive cheerleading routines comprise intermittent, short bursts of high-intensity effort, highlighting the anaerobic nature of the sport. Thus, in addition to technical proficiency, substantial muscular strength and power are essential to successfully execute these activities. The physical demands of cheerleading are further intensified by high injury rates, particularly involving the knees and ankles [3,4]. Muscular strength not only enhances performance but is also crucial to preventing injury. Current evidence suggests that skill-based, sport-specific training alone is insufficient to develop the strength and power necessary for elite-level cheerleading [5]. Hence, the integration of structured strength and conditioning (S&C) programs is essential to meet the physical standards required for high-level performance and long-term health of athletes.

In sports that require high levels of strength and power, such as Olympic weightlifting, periodized resistance training has been widely implemented [6]. These training approaches, typically progressing from hypertrophy-oriented phases to strength and power development, are considered crucial for optimizing performance in activities involving explosive and high-intensity movements [7,8,9]. However, despite the similar physical demands of cheerleading, the application and effectiveness of structured resistance training programs in this population remain poorly understood.

Several studies have outlined the general fitness characteristics of cheerleaders, reporting high levels of anaerobic capacity and favorable body composition [10,11]. A recent study reported that application of the Optimum Performance Training model developed by the National Academy of Sports Medicine within a hybrid cheerleading education context improved general physical fitness and movement efficiency compared to skill-based practice alone [12]. However, although research on cheerleading has expanded over the past two decades, most published work has primarily focused on injury epidemiology, safety guidelines, and descriptive practice characteristics rather than structured physical development. In particular, studies involving elite-level collegiate cheerleaders remain scarce, making it difficult to establish reference values for strength and power capabilities required for high-level performance and injury resilience. Moreover, Jacobson et al. [13] reported that although 92.9% of collegiate cheerleaders participate in weight training as an integral part of preparation, the effectiveness of specific training protocols remains insufficiently documented in the literature. To date, little is known about the types of effective S&C programs for this population and the specific adaptations resulting from periodized resistance training among elite collegiate cheerleaders.

Therefore, this study aimed to examine the effects of a 16-week periodized resistance training program on the strength, power, and body composition of elite collegiate cheerleaders, focusing on athletes in the base, spotter, and middle positions who frequently engage in lifting movements. The findings are expected to contribute to the development of preliminary performance benchmarks and training guidelines to support enhanced athletic performance and injury prevention of competitive cheerleading athletes.

## 2. Materials and Methods

### 2.1. Study Approval and Participant Consent

The study protocol was approved by Teikyo University Medical Research Ethics Committee (approval code: 2024-12) and conducted in accordance with the ethical principles for medical research involving human subjects described in the Declaration of Helsinki. Prior to inclusion in this study, informed consent was obtained from all participants.

### 2.2. Participants

The study cohort consisted of 13 female collegiate cheerleaders (mean values: age, 19.7 ± 0.9 years; height, 158.2 ± 4.0 cm; body mass, 58.8 ± 4.4 kg; sports participation, 13.1 ± 3.2 years; resistance training experience, 1.4 ± 0.5 years) from a nationally top-ranked collegiate team. The training intervention was conducted from January to June 2023. In the preceding season (2022), the team won both the All-Japan Cheerleading Championship and the All-Japan Collegiate Championship, reinforcing their status as the leading university program in Japan. The study specifically included athletes serving in base, spotter, and middle positions—roles that predominantly involve lifting, catching, and stabilizing during partner stunts and pyramid formation. Athletes in the top position were excluded because of their distinct anthropometric requirement of low body mass being advantageous for performance [5] and the potential for unintended weight gain during the hypertrophy phase of the intervention. All participants had a minimum of one year of prior resistance training experience and were actively engaged in cheerleading practice during the study period. The inclusion criteria required athletes to be healthy, free of musculoskeletal injuries at baseline, and consistently participate in team training. Athletes who sustained injuries during the intervention were excluded from the final analysis. Notably, no injuries occurred during the study period.

### 2.3. Study Design

This study employed a single-group longitudinal pre–post-design conducted over a 16-week formal intervention period. The overall periodized training program spanned 22 weeks and included a two-week preparatory familiarization phase at the outset and a three-week peaking phase leading up to a regional cheerleading competition in June 2023. An overview of the complete 22-week periodized resistance training program, including the training phases, scheduled events, and weekly session frequency, is presented in Table 1.

The primary 16-week intervention phase lasted from Week 3 to Week 18, during which outcome measures were collected at three time points: pre-intervention (Week 3), mid-intervention (Week 9), and post-intervention (Week 18). This timeframe was used to evaluate changes to strength, power, and body composition in response to the intervention. The program was structured into four progressive phases—familiarization, hypertrophy, strength/power, and peaking—based on established principles of periodization [9]. This periodization model—progressing from general to specific physical capacities—is aligned with evidence-based principles of physiological sequencing, in which hypertrophic adaptations establish a structural base for later neural and explosive training [8]. The training and testing schedules were carefully aligned with the team’s annual calendar, accounting for scheduled rest periods, training camps, academic semester start dates, and competition events, ensuring feasibility within the collegiate sports context. Although athletes continued their regular team practices and their dietary habits were not controlled as part of the study, the resistance training program was implemented in a structured and consistent manner under the supervision of the coaching staff to ensure uniform training exposure across participants and consistency throughout the intervention period.

### 2.4. Training Protocol

#### 2.4.1. Familiarization Phase (Weeks 1–2)

The familiarization phase aimed to promote neuromuscular adaptation and technical proficiency in foundational resistance training exercises. Participants trained using light to moderate loads (<80% of previous one-repetition maximum [1RM]) and performed movements included in subsequent phases. The exercise selection and intensity were tailored to the strength level and movement quality of each participant. This preparatory stage was designed to reduce the risk of early-phase injury and enable a safe and efficient transition to higher training volumes and intensities.

#### 2.4.2. Hypertrophy Phase (Weeks 3–11)

The hypertrophy phase focused on increasing skeletal muscle mass to support future strength gains. Training was conducted three times per week at moderate-to-high intensity (approximately 70–90% 1RM or 12–5RM) with moderate-to-high volume (3–6 sets of 5–10 repetitions). Each session included compound lifts, such as front squats, Bulgarian squats, and Olympic weightlifting variations (e.g., hang high pull and power clean), along with core and accessory work. A structured outline of the weekly training content is provided in Table 2.

#### 2.4.3. Strength and Power Phase (Weeks 12–19)

This phase emphasized the development of maximal strength and explosive power. Training intensity ranged from approximately 70% to 95% 1RM (12–3RM), with a reduction in training volume (3–5 sets of 3–10 repetitions) and frequency (twice weekly) to accommodate academic demands. Power clean, push press, and heavy squats were emphasized to stimulate neuromuscular adaptations. The detailed programming content is presented in Table 3.

#### 2.4.4. Peaking Phase (Weeks 20–22)

During the final three weeks, the participants followed a tapering protocol to reduce the training volume and optimize performance for subsequent competition. Although exercises and loads were not standardized, all athletes were instructed to include at least one power-type movement (e.g., power clean) at moderate to high intensity (>80% 1RM) for 3–5 sets per session. Training was self-regulated based on the physical condition of each athlete, with the primary goal of maintaining neuromuscular readiness.

### 2.5. Session Duration, Intensity Regulation, and Supervision

Training sessions typically lasted approximately 80 min, except during the peaking phase, which was shortened to approximately 30 min to prioritize recovery and readiness for competition. All sessions began with a standardized dynamic warm-up, followed by structured resistance training. The intensity was prescribed in terms of repetition maximum (RM). For instance, a session prescribed as “Front Squat/3 sets 5 reps/7–5RM” required athletes to choose a load that could be completed for 5–7 maximal repetitions. This system allowed autoregulation based on daily readiness, while minimizing technique breakdown and overtraining. Rest intervals ranged from 90 to 150 s depending on the load and the physical condition of the athlete. All sessions were supervised by a certified S&C specialist (National Strength and Conditioning Association) who provided real-time feedback and adjusted the training loads in response to fatigue or form deterioration.

### 2.6. Outcome Measures

All outcome variables were assessed at three time points aligned with the training schedule described in Table 1: pre-intervention (Week 3), mid-intervention (Week 9), and post-intervention (Week 18). Strength and power tests were administered at the beginning of each testing session following a standardized warm-up. Non-tested exercises were performed after the completion of all measurements. Body composition was assessed during the same week under controlled and standardized conditions.

#### 2.6.1. Strength and Power Test

Maximal muscular strength and explosive power were assessed using 1RM tests for the front squat, power clean, and shoulder press. These exercises were chosen to represent lower-body strength (front squat), lower-body power (power clean), and upper-body strength (shoulder press). The power clean is recognized as an effective movement to elicit high power output and has been associated with explosive performance in sprinting and jumping [14,15]. Accordingly, the 1RM power clean was used in this study as a field-based indicator of power-oriented performance rather than a direct measure of mechanical power.

To ensure measurement reliability, all tests were conducted under standardized conditions and supervised by a certified S&C specialist. Regular training was provided to ensure that the participants were familiar with the testing procedures. Similar 1RM testing protocols have demonstrated high test–retest reliability in resistance-trained populations (intraclass correlation coefficients [ICC] ≥ 0.90) [16]. Strict technical execution criteria were applied across all lifts. For the front squat, participants unracked an appropriate barbell from a squat rack onto the front of the shoulders, stepped back, descended until the thighs reached at least a parallel position to the floor, and then returned to a fully upright stance before re-racking. For the shoulder press, each attempt began from a fully locked-out overhead position while standing upright, lowering the bar below chin level, and pressing the bar back to full lockout without any contribution from the lower body. For the power clean, an appropriate barbell was lifted from the floor primarily via lower limb extension, accelerating the barbell vertically, and catching the barbell on the shoulders above the thigh-parallel level. Only attempts that satisfied the prescribed range of motion and positional standards were counted as valid.

All testing followed standardized procedures, including a structured warm-up, progressive load increments of 2.5–10 kg based on proximity to maximum, self-selected rest intervals between attempts (typically 90–150 s), and the use of Olympic lifting barbells. Testing was terminated after two consecutive unsuccessful attempts or earlier if the S&C specialist judged continued attempts unsafe due to a technical breakdown.

#### 2.6.2. Body Composition Test

Body composition was measured using a multi-frequency bioelectrical impedance analysis (BIA) device (InBody 770; InBody Co., Ltd., Tokyo, Japan). The measured variables included body mass, lean body mass, and body fat percentage. All measurements were performed in the morning under standardized conditions, with participants in a fasted state, to minimize the potential influence of diurnal variation and recent nutritional intake. Multi-frequency BIA devices, including the InBody 770, have demonstrated excellent test–retest reliability under controlled measurement conditions, with ICC ≥ 0.98 [17].

### 2.7. Statistical Analysis

All statistical analyses were conducted using IBM SPSS Statistics version 29.0.1.0 (IBM Corp., Armonk, NY, USA). Descriptive statistics are presented as the mean ± standard deviation. For each outcome at each time point, normality was examined using the Shapiro–Wilk test to select appropriate subsequent analytical procedures. The analyzed outcomes included 1RM expressed relative to body mass (% body mass) for the front squat, power clean, and shoulder press, as well as body mass (kg), lean body mass (kg), and body fat percentage (%). Relative 1RM values were used to account for differences in body size and facilitate meaningful comparisons across studies and applied settings. Moreover, body composition variables were analyzed using their most appropriate forms, with lean body mass expressed in absolute terms to reflect changes in muscle mass and body fat expressed as a percentage to represent relative fatness. When data met normality assumptions, changes across the three measurement points (pre-, mid-, and post-intervention) were examined using one-way repeated-measures analysis of variance (ANOVA). If a significant main effect was detected, Bonferroni-adjusted pairwise comparisons were performed to control for type I errors. For variables violating normality assumptions, the Friedman test was used as the nonparametric alternative. When significant, post hoc pairwise comparisons were conducted using the Wilcoxon signed-rank test with Bonferroni correction applied by multiplying each probability (*p*) value by the number of comparisons. The effect size for pairwise contrasts was calculated using Hedges’ *g* (bias-corrected for small samples) and interpreted according to Hopkins [18]: *g* < 0.20 = trivial, 0.20–0.59 = small, 0.60–1.19 = moderate, 1.20–1.99 = large, 2.00–3.99 = very large, and ≥4.00 = extremely large. Statistical significance was set at *p* < 0.05. A post hoc power analysis was conducted using G*Power (version 3.1.9.7) for the repeated-measures ANOVA design. Based on the observed effect sizes for the primary outcomes (e.g., body fat percentage), with an alpha of 0.05 and a sample size of 13 across three measurement time points, the achieved statistical power exceeded 0.99.

## 3. Results

The normality of all data was verified (*p* > 0.05), except for body mass at the pre- (*p* = 0.022), mid- (*p* = 0.006), and post (*p* = 0.006)-time points, and for lean body mass at the pre- (*p* = 0.033) and mid (*p* = 0.039)-time points.

### 3.1. 1RM Outcomes

**Figure 1:**
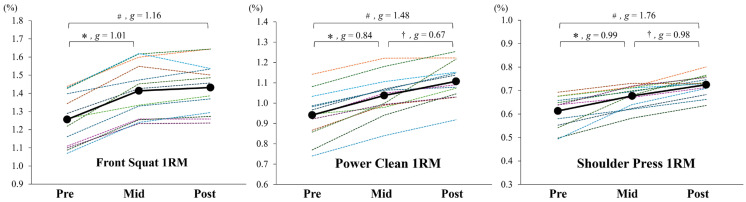
Individual (dashed lines) and group mean (solid lines) changes to one-repetition maximum (1RM) values relative to the body mass for front squat, power clean, and shoulder press across the pre-, mid-, and post-intervention time points. * Significantly different between pre and mid (all, *p* < 0.001); † between mid and post (*p* = 0.002 for power clean, *p* < 0.001 for shoulder press); and # between pre and post (all, *p* < 0.001). Effect sizes are presented as Hedges’ *g* for each pairwise comparison. Comparisons with non-significant and trivial effects (*g* < 0.2, *p* > 0.05) were omitted for clarity.

All 1RM outcomes significantly improved over the course of the intervention (Figure 1, Table 4). Repeated-measures ANOVA revealed significant main effects of time on 1RM values relative to body mass for the front squat (*p* < 0.001, partial η^2^ = 0.909), power clean (*p* < 0.001, partial η^2^ = 0.820), and shoulder press (*p* < 0.001, partial η^2^ = 0.793). Post hoc comparisons indicated that from pre- to mid-intervention, 1RM values significantly increased for all three exercises (*p* < 0.001), with moderate effect sizes (*g* = 0.84–1.01). From mid- to post-intervention, additional significant increases in 1RM values were observed for the power clean (*p* = 0.002) and shoulder press (*p* < 0.001), both with moderate effect sizes (*g* = 0.67–0.98), whereas no significant change was observed for the front squat (*p* = 0.446, *g* = 0.12). From pre- to post-intervention, all exercises demonstrated statistically significant improvements in 1RM values (all, *p* < 0.001), with moderate to large effect sizes (*g* = 1.16–1.76).

### 3.2. Body Composition

**Figure 2:**
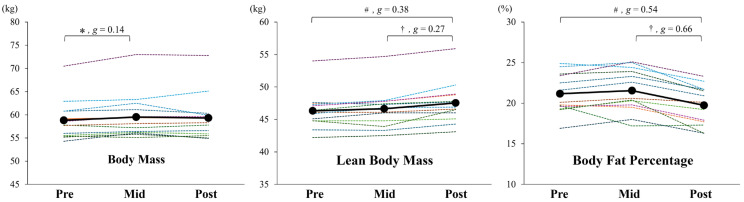
Individual (dashed lines) and group mean (solid lines) changes to body mass, lean body mass, and body fat percentage across the pre-, mid-, and post-intervention time points. * Significantly different between pre and mid (*p* = 0.036); † between mid and post (*p* = 0.006 for lean body mass, *p* < 0.001 for body fat percentage); and # between pre and post (*p* = 0.003 for lean body mass, *p* = 0.001 for body fat percentage). Effect sizes (Hedges’ *g*) are reported for each pairwise comparison. Comparisons with non-significant and trivial effects (*g* < 0.2, *p* > 0.05) are not shown for clarity.

Significant changes were observed in multiple body composition variables during the intervention (Figure 2, Table 4).

For body mass, the assumption of normality was not met; thus, a nonparametric approach was applied. The Friedman test indicated a significant main effect of time (*p* = 0.027, Kendall’s W = 0.278). Post hoc comparisons with the Wilcoxon signed-rank test revealed a significant increase from pre- to mid-intervention (Bonferroni-adjusted *p* = 0.036, *g* = 0.14), whereas there was no significant difference from mid- to post-intervention (adjusted *p* = 1.437, *g* = 0.04) or from pre- to post-intervention (adjusted *p* = 0.231, *g* = 0.10).

Similarly, for lean body mass, normality was not confirmed. The Friedman test demonstrated a significant main effect (*p* < 0.001, Kendall’s W = 0.783). The Wilcoxon signed-rank test showed no significant change from pre- to mid-intervention (adjusted *p* = 0.204, *g* = 0.10) but a significant increase from mid- to post-intervention (adjusted *p* = 0.006, *g* = 0.27) and from pre- to post-intervention (adjusted *p* = 0.003, *g* = 0.38).

For body fat percentage, normality assumptions were satisfied; therefore, repeated-measures ANOVA was applied. The analysis revealed a significant main effect of time (*p* < 0.001, partial η^2^ = 0.633). Post hoc analyses showed no significant change from pre- to mid-intervention (*p* = 0.646, *g* = 0.14), but a significant reduction from mid- to post-intervention with a moderate effect (*p* < 0.001, *g* = 0.66) and a significant reduction from pre- to post-intervention with a small effect (*p* = 0.001, *g* = 0.54).

## 4. Discussion

This study aimed to examine the effects of a 16-week periodized resistance training program on the muscular strength, power, and body composition of elite collegiate cheerleaders. Overall, all three 1RM measures (front squat, power clean, and shoulder press) demonstrated meaningful improvements from pre- to post-intervention (Figure 1, Table 4). Favorable adaptations were also observed in lean body mass and body fat percentage, whereas total body mass remained unchanged (Figure 2, Table 4), indicating that strength, power, and body composition had improved without an unwanted increase in body weight—a desirable outcome, given the technical and positional demands of cheerleading. Collectively, these findings suggest the potential value of structured S&C programming for this population and contribute to defining preliminary strength and body composition benchmarks for high-performance collegiate cheerleaders.

The time course of adaptation provides additional insights into how cheerleaders may respond to long-term resistance training. Notably, the largest strength gains occurred during the initial hypertrophy-focused phase, while improvements to body composition—such as increased lean mass and reduced fat percentage—were more pronounced during the later strength/power phase (Figure 1 and Figure 2, Table 4), although these findings should be interpreted with caution given the use of a BIA device, as its findings might be influenced by physiological factors, including variations in hydration status, potentially affecting the accuracy of body composition estimates [19]. While this sequence does not align perfectly with conventional expectations that hypertrophy emerges early and strength peaks later, it is nevertheless consistent with the established physiological models of adaptation. Specifically, early-phase strength gains are often driven by neural adaptations, such as enhanced motor unit recruitment [20], whereas morphological changes, such as increases in muscle cross-sectional area, tend to emerge more gradually, often requiring 6 to 10 weeks of sustained mechanical loading and accumulated training volume [21,22]. Similar deviations from planned phase outcomes have been observed in other athlete populations. For instance, Wetmore et al. [23] reported that less-trained individuals often respond most during early training blocks, while more advanced athletes show greater improvements later, suggesting that training adaptations do not always follow a fixed schedule and may vary depending on training history, recovery status, and cumulative effects across phases. As such, while periodization provides a valuable framework, coaches should remain flexible in interpreting outcomes and adjusting programs based on observed responses.

Across the 16-week intervention, the 1RM values of the front squat, power clean, and shoulder press increased by +14.0%, +17.7%, and +18.3%, respectively (Table 4). Although these findings should be interpreted in consideration of potential sources of bias and influences such as regular cheerleading practice and nutritional intake, the magnitude of these improvements appears substantial for athletes with prior experience in resistance training [24]. Importantly, the post-intervention strength levels observed in the present study were comparable to, and in several cases exceeded, previously reported reference values of high-performance female athletes. For example, the front squat value of 1.43× body mass surpassed reference levels reported for elite female field hockey athletes (1.28× body mass) [25]. Considering that back squat strength ≥1.60× body mass has been suggested as a criterion associated with performance and injury resilience of collegiate female athletes [26], and given the approximate front squat-to-back squat 1RM ratio of 84% [27], a front squat reference standard of ~1.30× body mass would represent a comparable benchmark. The athletes in this study exceeded this threshold, suggesting a lower-body strength profile consistent with levels associated with performance enhancement and potentially related to injury reduction. Upper-body strength outcomes were also noteworthy, with the shoulder press value of 0.73× body mass, which is comparable to reported averages of competitive CrossFit athletes (0.71× body mass) [28]. Although data on relative power clean performance of female athletes remain limited, the available literature suggests values of 74% to 83% of body mass for trained populations [29,30]. Conversely, the present value of 1.11× body mass substantially exceeded these norms, highlighting a notably high capacity for explosive-oriented performance. Body composition findings (19.7 ± 2.4%) were comparable to prior reports, as the body fat percentage of elite cheerleaders reportedly ranges from ~15% to 22% [10,11]. This value also falls within the commonly observed range for elite female athletes participating in volleyball, soccer, and basketball (~19–22%) [31].

Collectively, these comparisons suggest that the athletes in the present study demonstrated strength, power, and body composition characteristics that are equal to or exceed those reported for females participating in high-performance collegiate sports. These outcomes reinforce the classification of collegiate cheerleading as a physically demanding, strength-dependent sport and highlight the potential performance benefits of systematic S&C programming in this population. Furthermore, the strength values reported in the study may serve as preliminary reference values to evaluate physical readiness and training targets for athletes performing lifting roles, such as the base, middle, and spotter positions.

### 4.1. Practical Implications

The findings of this study provide several practical implications for S&C coaches working with collegiate cheerleaders. Firstly, the results suggest that structured periodized resistance training might contribute to improvements in strength and body composition without increasing total body mass, which is particularly relevant to the physical and technical demands of cheerleading. Secondly, this training program might provide a practical example for designing resistance training interventions in similar populations, particularly in terms of phase structure and progression from hypertrophy to strength and power development. Thirdly, the relative strength values and body composition characteristics observed in this study could represent preliminary reference values to guide performance evaluation and goal setting in applied settings. Finally, the observed variability in the time course of adaptation highlights the importance of flexible program design and ongoing monitoring to optimize individual responses to training.

### 4.2. Limitations and Future Perspectives

This study had some limitations. Firstly, the sample was drawn from a single collegiate cheerleading program, and the sample size was relatively small, which might limit generalizability to other teams, competitive levels, and age groups. Additionally, although the roster included athletes in the base, middle, and spotter positions, top athletes were not represented in the sample. However, because strength improvements were achieved without increasing total body mass, the training approach described herein may also be applicable to top athletes who rely on aerial performance. Secondly, as a single-group longitudinal pre–post design was used, it is not possible to attribute the observed changes exclusively to the training intervention. External factors, such as regular cheerleading practice, nutritional intake, and general training adaptations over time, might have contributed to the observed outcomes. Therefore, the results should be interpreted cautiously, and causal inferences regarding the effectiveness of the intervention were not definitively established. Thirdly, body composition was assessed using BIA, and despite its practical nature and wide use in applied settings, the analysis might have been affected by factors such as hydration status and recent nutritional intake [19]. However, to minimize these potential influences, all measurements were conducted under standardized conditions, including consistent assessment timing and a fasted state. Future research incorporating controlled study designs, longitudinal athlete monitoring, and positional subgroup analyses—including top athletes—could further strengthen the understanding of training responses in cheerleading.

## 5. Conclusions

This study found that a 16-week periodized resistance training program was associated with improvements in the strength, power, and body composition of elite collegiate cheerleaders without increasing total body mass. The improvements observed across key strength measures and physiological characteristics suggest that cheerleaders, even those with prior training experience, might adapt positively to structured resistance training. These findings provide a foundation for the development of evidence-based training guidelines and preliminary strength benchmarks for collegiate cheerleading. Continued research across broader populations, positions, and competitive levels can help refine these recommendations and further support long-term athlete development in the sport.

## Figures and Tables

**Table 1:** Overview of the 22-week periodized resistance training schedule, indicating training phases, special events, and weekly training session frequencies. Outcome measures were collected at pre-, mid-, and post-testing during the 16-week formal intervention period (Weeks 3–18).

Week	Event	Training Phase	Sessions/Week
1		Familiarization	3
2		Familiarization	3
3	Pre-Test	Hypertrophy	3
4		Hypertrophy	3
5		Hypertrophy	3
6	Team Rest		0
7		Hypertrophy	3
8		Hypertrophy	3
9	Mid-Test	Hypertrophy	3
10	Training Camp		0
11		Hypertrophy	3
12	Beginning of School	Strength/Power	2
13		Strength/Power	2
14		Strength/Power	2
15		Strength/Power	2
16		Strength/Power	2
17		Strength/Power	2
18	Post-Test	Strength/Power	2
19		Strength/Power	2
20		Peaking	2
21		Peaking	2
22	Competition	Peaking	1

**Table 2:** Training overview of the hypertrophy phase, outlining exercises, set/rep schemes, intensities, and frequencies.

Hypertrophy Phase											
Day 1				Day 2				Day 3			
Exercise	Sets	Reps	Intensity	Exercise	Sets	Reps	Intensity	Exercise	Sets	Reps	Intensity
Side Plank Variations	3	-	bodyweight	Front Plank Variations	3	-	bodyweight	Side Plank Variations	3	-	bodyweight
Bulgarian Squat	3	10 each	12–10RM	Hang High Pull	5	10	12–10RM	Pull Up	4	10	bodyweight
Pin Front Squat	6	10	12–10RM	Romanian Deadlift	3	10	12–10RM	Bulgarian Squat	3	10 each	12–10RM
Power Clean	5	5	7–5RM	Power Clean & Push Press	5	5	7–5RM	Front Squat	2	10	12–10RM
Shoulder Press	4	10	12–10RM	Bar Hanging + Leg Raise	3	15 s +10	bodyweight		1	7	9–7RM
Inverted Row	4	10	bodyweight						2	5	7–5RM
Ab Roller	3	10	bodyweight					Goblet Squat	3	10	12RM

RM—repetition maximum.

**Table 3:** Training overview of the strength and power phases, outlining exercises, set/rep schemes, intensities, and frequencies.

Strength and Power Phase							
Day 1				Day 2			
Exercise	Sets	Reps	Intensity	Exercise	Sets	Reps	Intensity
Ab Roller	3	10	Bodyweight	Ab Roller	3	10	Bodyweight
Bulgarian Squat	3	10 each	12–10RM	Bulgarian Squat	3	10 each	12–10RM
Power Clean & Push Press	5	5	7–5RM	Pin Front Squat	5	10	12–10RM
Front Squat	1	8	10–8RM	Power Clean	5	5	7–5RM
	1	6	8–6RM		3	3	5–3RM
	1	4	6–4RM	Single Leg Hip Thrust	3	10 each	Bodyweight
	2	3	3RM	Back Extension	3	15	Bodyweight
Shoulder Press	4	5	7–5RM				
Scapular Pull Up + Bent Over Row	4	10 + 10	Bodyweight				

RM—repetition maximum.

**Table 4:** Descriptive statistics (mean ± standard deviation) and percentage changes (%Δ) in one-repetition maximum values relative to body mass and body composition variables at pre-, mid-, and post-intervention. %Δ values represent the relative change from the earlier time point: pre–mid, mid–post, and pre–post.

Variable	Pre	Mid	Post	%Δ Pre–Mid	%Δ Mid–Post	%Δ Pre–Post
One-Repetition Maximum						
Front Squat (% body mass)	1.26 ± 0.14	1.41 ± 0.15	1.43 ± 0.14	+12.6%	+1.3%	+14.0%
Power Clean (% body mass)	0.94 ± 0.11	1.04 ± 0.10	1.11 ± 0.09	+10.3%	+6.7%	+17.7%
Shoulder Press (% body mass)	0.61 ± 0.07	0.68 ± 0.05	0.73 ± 0.04	+10.4%	+7.1%	+18.3%
Body Composition						
Body Mass (kg)	58.8 ± 4.4	59.5 ± 4.8	59.3 ± 4.9	+1.2%	−0.4%	+0.8%
Lean Body Mass (kg)	46.3 ± 2.8	46.6 ± 3.0	47.5 ± 3.2	+0.7%	+1.9%	+2.6%
Body Fat Percentage (%)	21.2 ± 2.4	21.5 ± 2.7	19.7 ± 2.4	+1.8%	−8.4%	−6.7%

## Data Availability

Data are available from the corresponding author upon reasonable request.

## References

[1] Shields B.J., Fernandez S.A., Smith G.A. (2009). Epidemiology of Cheerleading Stunt-Related Injuries in the United States. J. Athl. Train..

[2] Riddell S., Zinner C., Lubiak S.M., Tiralla G., Foster T., Tamulevicius N., Quittmann O.J., Lange M., Gavanda S. (2025). Physiological Responses of Elite Cheerleaders During Training and Simulated Competition Routines. Int. J. Sports Physiol. Perform..

[3] Bagnulo A. (2012). Cheerleading Injuries: A Narrative Review of the Literature. J. Can. Chiropr. Assoc..

[4] LaBella C.R., Mjaanes J., Council on Sports Medicine and Fitness (2012). Cheerleading Injuries: Epidemiology and Recommendations for Prevention. Pediatrics.

[5] Goodwin E.P., Adams K.J., Shelburne J., Debeliso M. (2004). A Strength and Conditioning Model for a Female Collegiate Cheerleader. Natl. Strength Cond. Assoc..

[6] Stone M.H., Pierce K.C., Sands W.A., Stone M.E. (2006). Weightlifting: A Brief Overview. Strength Cond. J..

[7] Stone M.H., Pierce K.C., Sands W.A., Stone M.E. (2006). Weightlifting: Program Design. Strength Cond. J..

[8] Stone M.H., O’Bryant H., Garhammer J., McMillan J., Rozenek R. (1982). A Theoretical Model of Strength Training. Natl. Strength Coach. Assoc. J..

[9] Bompa T.O., Buzzichelli C. (2019). Periodization: Theory and Methodology of Training.

[10] Thomas D.Q., Seegmiller J.G., Cook T.L., Young B.A. (2004). Physiologic Profile of the Fitness Status of Collegiate Cheerleaders. J. Strength Cond. Res..

[11] Bellissimo M.P., Licata A.D., Nucci A., Thompson W., Benardot D. (2019). Relationships Between Estimated Hourly Energy Balance and Body Composition in Professional Cheerleaders. J. Sci. Sport Exerc..

[12] Yu J. (2025). Optimizing NASM-OPT Training in Hybrid Cheerleading Education: A Biomechanical Approach. Mol. Cell. Biomech..

[13] Jacobson B.H., Redus B., Palmer T. (2005). An Assessment of Injuries in College Cheerleading: Distribution, Frequency, and Associated Factors. Br. J. Sports Med..

[14] Hori N., Newton R.U., Andrews W.A., Kawamori N., McGuigan M.R., Nosaka K. (2008). Does Performance of Hang Power Clean Differentiate Performance of Jumping, Sprinting, and Changing of Direction?. J. Strength Cond. Res..

[15] Suchomel T.J., Comfort P., Lake J.P. (2017). Enhancing the Force-Velocity Profile of Athletes Using Weightlifting Derivatives. Strength Cond. J..

[16] Grgic J., Lazinica B., Schoenfeld B.J., Pedisic Z. (2020). Test–Retest Reliability of the One-Repetition Maximum (1RM) Strength Assessment: A Systematic Review. Sports Med. Open.

[17] McLester C.N., Nickerson B.S., Kliszczewicz B.M., McLester J.R. (2020). Reliability and Agreement of Various InBody Body Composition Analyzers as Compared to Dual-Energy X-Ray Absorptiometry in Healthy Men and Women. J. Clin. Densitom..

[18] Hopkins W.G., Marshall S.W., Batterham A.M., Hanin J. (2009). Progressive Statistics for Studies in Sports Medicine and Exercise Science. Med. Sci. Sports Exerc..

[19] Potter A.W., Ward L.C., Chapman C.L., Tharion W.J., Looney D.P., Friedl K.E. (2025). Real-World Assessment of Multi-Frequency Bioelectrical Impedance Analysis (MFBIA) for Measuring Body Composition in Healthy Physically Active Populations. Eur. Nutr..

[20] Moritani T., deVries H.A. (1979). Neural Factors versus Hypertrophy in the Time Course of Muscle Strength Gain. Am. J. Phys. Med..

[21] Wernbom M., Augustsson J., Thomeé R. (2007). The Influence of Frequency, Intensity, Volume and Mode of Strength Training on Whole Muscle Cross-Sectional Area in Humans. Sports Med..

[22] Lecce E., Amoruso P., Felici F., Bazzucchi I. (2025). Resistance Training-induced Adaptations in the Neuromuscular System: Physiological Mechanisms and Implications for Human Performance. J. Physiol..

[23] Wetmore A.B., Moquin P.A., Carroll K.M., Fry A.C., Hornsby W.G., Stone M.H. (2020). The Effect of Training Status on Adaptations to 11 Weeks of Block Periodization Training. Sports.

[24] Zouita A., Darragi M., Bousselmi M., Sghaeir Z., Clark C.C.T., Hackney A.C., Granacher U., Zouhal H. (2023). The Effects of Resistance Training on Muscular Fitness, Muscle Morphology, and Body Composition in Elite Female Athletes: A Systematic Review. Sports Med..

[25] Ransdell L.B., Murray T. (2011). A Physical Profile of Elite Female Ice Hockey Players from the USA. J. Strength Cond. Res..

[26] Case M.J., Knudson D.V., Downey D.L. (2020). Barbell Squat Relative Strength as an Identifier for Lower Extremity Injury in Collegiate Athletes. J. Strength Cond. Res..

[27] Ray T., Adams K.J., DeBeliso M. (2017). The Relationship Between Core Stability & Squat Ratio in Resistance-Trained Males. Int. J. Kinesiol. Sports Sci..

[28] Menargues-Ramírez R., Sospedra I., Holway F., Hurtado-Sánchez J.A., Martínez-Sanz J.M. (2022). Evaluation of Body Composition in CrossFit^®^ Athletes and the Relation with Their Results in Official Training. Int. J. Environ. Res. Public Health.

[29] Bruenger A. Squat and Power Clean Strength Are Not Related to Appropriate Drop Jump Height in Female Collegiate Athletes. Proceedings of the 32nd International Conference on Biomechanics in Sports (ISBS).

[30] Comfort P., McMahon J.J., Fletcher C. (2013). No Kinetic Differences During Variations of the Power Clean in Inexperienced Female Collegiate Athletes. J. Strength Cond. Res..

[31] Mala L., Maly T., Zahalka F., Bunc V., Kaplan A., Jebavy R., Tuma M. (2015). Body Composition of Elite Female Players in Five Different Sports Games. J. Hum. Kinet..

